# Does Anemia Independently Influence the Erythrocyte Sedimentation Rate in Rheumatoid Arthritis? A Multivariate Analysis From North India

**DOI:** 10.7759/cureus.104704

**Published:** 2026-03-05

**Authors:** Ashish Jindal, Kiranpreet Kaur, Ashish Goel, Parteek Setia, Saurabh Lanjewar

**Affiliations:** 1 Department of General Medicine, Dr. B.R. Ambedkar State Institute of Medical Sciences Mohali, Sahibzada Ajit Singh Nagar, IND

**Keywords:** anemia, c-reactive protein, erythrocyte sedimentation rate, multivariate analysis, north india, rheumatoid arthritis, seropositivity

## Abstract

Background: In regions such as North India, where anemia is prevalent, interpretation of the erythrocyte sedimentation rate (ESR) in rheumatoid arthritis (RA) may be challenging. We evaluated the independent associations of inflammation, autoantibody status, and anemia with ESR elevation and examined morphologic anemia patterns in this cohort.

Methods: We conducted a retrospective cross-sectional analysis of 152 RA patients attending a tertiary care center. Multivariate logistic regression identified independent predictors of elevated ESR and of anemia after adjustment for covariates. Seropositivity was defined by rheumatoid factor (RF) or anti-cyclic citrullinated peptide (anti-CCP) positivity. Among anemic patients, anemia was classified morphologically as microcytic or normocytic based on mean corpuscular volume (MCV), and inflammatory markers were compared across groups. Missing laboratory data were addressed using multiple imputation by chained equations (MICE).

Results: The cohort was predominantly female (133/152, 87.5%), and seropositive RA accounted for 75.7% (115/152) of cases. In the ESR model, C-reactive protein (CRP) positivity was the strongest independent predictor (adjusted odds ratio [aOR] 7.81, p<0.001), whereas anemia was not independently associated after adjustment (aOR 1.45, p=0.305). In a separate model predicting anemia, seropositive RA remained independently associated (aOR 2.12, p=0.024). Among anemic patients (n=89), 87 had available morphologic data; of these, 47 (54%) were normocytic and 40 (46%) microcytic. The normocytic group demonstrated significantly higher ESR and CRP values than other groups (p<0.001).

Conclusion: ESR elevation was independently associated with CRP-defined inflammatory activity after multivariate adjustment, whereas anemia did not demonstrate an independent association. These findings suggest that ESR retains clinical relevance in reflecting inflammatory burden when interpreted in conjunction with complementary laboratory parameters.

## Introduction

Rheumatoid arthritis (RA) is a systemic autoimmune disease that imposes a significant and growing public health burden in India, with a prevalence of approximately 0.75% and projections suggesting an increasing disease impact through 2036 [1,2]. Although RA typically presents as symmetric polyarthritis, persistent systemic inflammation contributes to multiple extra-articular manifestations that may adversely affect long-term outcomes [3,4].

Serologically, RA is classified as seropositive (SPRA) when rheumatoid factor (RF) or anti-cyclic citrullinated peptide (anti-CCP) antibodies are present and seronegative (SNRA) when these autoantibodies are absent [5]. Seropositive RA has been associated with higher inflammatory burden and more aggressive structural progression in several cohorts [6,7].

Inflammatory markers such as erythrocyte sedimentation rate (ESR) and C-reactive protein (CRP) are routinely used in clinical practice to assess inflammatory activity in RA. However, interpretation of ESR may be complicated by coexisting hematologic factors, particularly anemia. Anemia is a common extra-articular manifestation of RA, reported in approximately 30-70% of patients, and may be especially frequent in populations with a high background prevalence of nutritional deficiency [8].

Reduced hemoglobin concentration can independently influence sedimentation dynamics, potentially elevating ESR values irrespective of inflammatory activity [9]. In RA, anemia is often described as either inflammation-associated (commonly normocytic) or iron-deficiency-related (commonly microcytic), although overlap between patterns is well recognized [6,10]. In routine clinical settings where iron parameters are not uniformly available, mean corpuscular volume (MCV) is frequently used as a pragmatic morphologic discriminator. For example, Agrawal et al. reported a high prevalence of anemia in Indian RA patients, with both normocytic and microcytic patterns commonly observed [11].

Although erythrocyte sedimentation rate (ESR) and C-reactive protein (CRP) are frequently used together as markers of inflammatory activity in rheumatoid arthritis, they reflect distinct biological mechanisms. CRP is a hepatically synthesized acute-phase reactant that responds rapidly to cytokine-mediated inflammatory signaling, whereas ESR is influenced not only by acute-phase proteins such as fibrinogen but also by erythrocyte characteristics, plasma composition, and hematologic parameters. In populations with a high background prevalence of anemia, interpretation of ESR becomes particularly complex because reduced hemoglobin concentration and altered red cell dynamics may independently affect sedimentation rates. Quantifying the relative contribution of inflammatory versus hematologic factors is therefore clinically relevant in routine rheumatology practice.

While aspects of these relationships have been examined individually, integrated multivariate analyses simultaneously evaluating inflammatory markers, serostatus, and morphologic anemia patterns within Indian RA cohorts remain limited [6,12]. The present study, therefore, aimed to (1) identify independent predictors of elevated ESR and of anemia using multivariate regression modeling; (2) examine inflammatory marker profiles across morphologically defined anemia patterns; and (3) compare selected clinical and laboratory characteristics between seropositive and seronegative RA in our cohort.

## Materials and methods

Study design and setting

We conducted a retrospective cross-sectional analysis of patients attending a rheumatology clinic at a tertiary care teaching hospital in North India between May 2022 and July 2025. All patients were evaluated by a rheumatologist, and diagnoses were established according to the 2010 American College of Rheumatology/European League Against Rheumatism (ACR/EULAR) classification criteria for rheumatoid arthritis (RA) [5].

The clinic database represents a prospectively maintained clinical registry used for routine patient management. Laboratory investigations were performed according to the treating physician's discretion rather than a standardized research protocol, which accounts for variability in the availability of certain parameters.

Eligibility criteria

**Figure 1:**
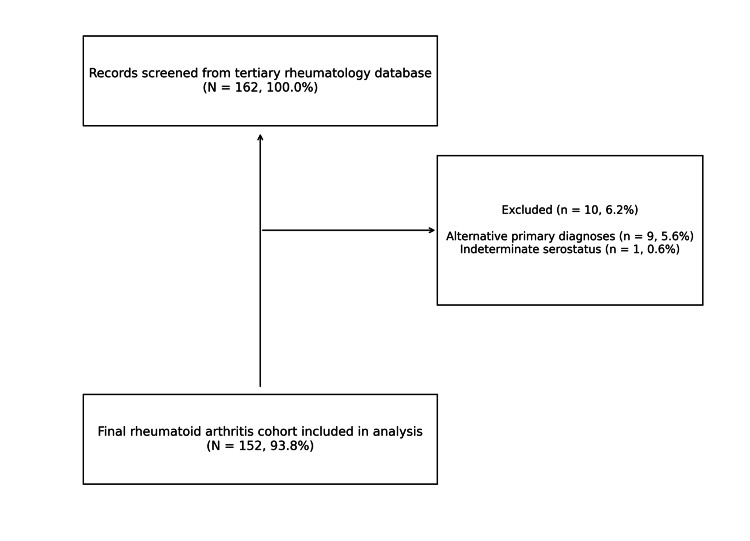
Patient Selection Flow Diagram Flow diagram illustrating screening and inclusion of patients from the tertiary rheumatology database. Of 162 records screened, 10 (6.2%) were excluded due to alternative primary diagnoses (n=9, 5.6%) or indeterminate serostatus (n=1, 0.6%), resulting in a final rheumatoid arthritis cohort of 152 (93.8%) included in the analysis. Data are presented as N (%).

Adults (≥18 years) with a confirmed diagnosis of RA and available baseline laboratory data were eligible for inclusion. Patients were excluded if (1) an alternative primary diagnosis was recorded (n=9) or (2) serostatus could not be determined due to the absence of both rheumatoid factor (RF) and anti-cyclic citrullinated peptide (anti-CCP) results (n=1). A total of 152 patients comprised the final analytical cohort. A flow diagram summarizing patient inclusion is presented in Figure 1.

Data collection and variable definitions

Data were extracted from an existing rheumatology clinic database. For each patient, only the earliest available laboratory panel at presentation to the rheumatology clinic was included to ensure independence of observations and was not standardized to flare or remission status. Both qualitative and quantitative laboratory reports were incorporated using predefined criteria to ensure consistent classification. Serial follow-up measurements were not analyzed in this cross-sectional study.

Serostatus Classification

Patients were classified as seropositive RA (SPRA) if RF or anti-CCP was positive. Patients negative for both rheumatoid factor (RF) and anti-cyclic citrullinated peptide (anti-CCP) antibodies were classified as seronegative RA (SNRA).

RF Positivity

A result was considered positive if qualitatively reported as positive or if the quantitative value exceeded 20 IU/mL [13,14].

Anti-CCP Positivity

A result was considered positive if qualitatively reported as positive or if the quantitative value exceeded 20 U/mL [15,16].

Inflammatory Markers

Erythrocyte sedimentation rate (ESR) positivity was determined using quantitative values according to age- and sex-adjusted Miller criteria [17]. C-reactive protein (CRP) positivity was defined as either a qualitative positive result or a quantitative value greater than 10 mg/L [18]. CRP was reported quantitatively (mg/L) in the majority of cases. In instances where CRP was documented categorically (positive/negative), classification was based on the institutional reference threshold of 10 mg/L. For analytical consistency, CRP was coded as positive when >10 mg/L.

Autoantibody testing was performed using standardized tertiary-care assays. IgM rheumatoid factor (RF) was measured via nephelometry (positivity threshold >20 IU/mL), and anti-cyclic citrullinated peptide (anti-CCP) antibodies were measured using a second-generation enzyme-linked immunosorbent assay (ELISA) (positivity threshold >20 U/mL). The laboratory reference threshold for C-reactive protein (CRP) elevation was 10 mg/L.

Hematologic Profile and Anemia Classification

Hemoglobin (Hb) was analyzed as a continuous variable. Anemia was defined according to World Health Organization criteria (Hb <12 g/dL in females and <13 g/dL in males) [19]. Among anemic patients, morphologic classification was performed using mean corpuscular volume (MCV) as microcytic (<80 fL), normocytic (80-100 fL), or macrocytic (>100 fL) [10]. No macrocytic anemia was observed in this cohort. These morphologic categories were defined for descriptive and comparative purposes and do not establish etiologic diagnoses.

Other Laboratory Variables

Serum creatinine, blood urea, serum glutamic-oxaloacetic transaminase (SGOT), and serum glutamic-pyruvic transaminase (SGPT) were analyzed as continuous variables.

All laboratory investigations were performed in the institutional central laboratory accredited under national quality standards. Hematologic parameters, including hemoglobin and mean corpuscular volume, were measured using an automated hematology analyzer calibrated and quality-checked according to manufacturer specifications and institutional laboratory protocols. ESR was measured using the Westergren method, and CRP was quantified using an immunoturbidimetric assay on an automated platform. Internal quality control procedures and routine instrument calibration checks were conducted daily, external quality assurance participation was maintained throughout the study period, and all reports were reviewed and validated by certified clinical pathologists.

Statistical analysis

Statistical analyses were performed using R software, version 4.3.2 (R Core Team, Vienna, Austria). A two-tailed p-value <0.05 was considered statistically significant. Continuous variables were summarized as mean ± standard deviation (SD) when normally distributed and as median with interquartile range (IQR) when non-normally distributed. These are presented in separate tables accordingly. Categorical variables were presented as numbers (percentages). Between-group comparisons were performed using Student’s t-test or Mann-Whitney U test for continuous variables and the chi-square test or Fisher’s exact test for categorical variables as appropriate. Spearman’s rank correlation was used to evaluate associations between continuous laboratory parameters.

Of the 152 patients included in the study, 99 had complete data across all core variables included in the primary multivariable models, whereas 53 had at least one missing laboratory parameter. Given this degree of missingness, multiple imputation by chained equations (MICE) was employed.

Missing data for selected laboratory variables (including mean corpuscular volume, ESR, and CRP) were handled using multiple imputation by chained equations (MICE). Twenty imputed datasets were generated, and pooled parameter estimates were derived using Rubin’s rules [20,21]. The imputation model incorporated all predictors and outcomes included in subsequent regression analyses. The missing at random (MAR) assumption was considered plausible in this clinical context, as the likelihood of laboratory testing was dependent on observed clinical characteristics. Patterns of missingness were evaluated descriptively, and sensitivity analyses were conducted using complete-case datasets to assess the robustness of the primary findings.

Multiple imputation was selected as the primary analytic strategy because complete-case restriction may introduce bias when laboratory testing is not missing completely at random. This approach preserves statistical power and is recommended in clinical epidemiology when missingness is plausibly dependent on observed variables.

To examine independent associations while adjusting for potential confounding, multivariate logistic regression analyses were conducted. In Model 1, anemia (yes/no) was specified as the dependent variable and included CRP positivity, serostatus (SPRA vs SNRA), age (per 10-year increase), and sex as predictor variables. In Model 2, ESR positivity (defined using age- and sex-adjusted Miller criteria [17]) was specified as the dependent variable and included CRP positivity, anemia status, age (per 10-year increase), and sex as predictors. Adjusted odds ratios (aORs) with 95% confidence intervals (CIs) were calculated based on pooled estimates from the multiply imputed datasets.

To evaluate differences across morphologically defined anemia categories, continuous inflammatory markers were compared using the Kruskal-Wallis test and categorical variables using the chi-square test.

Although logistic regression aligned with age- and sex-adjusted Miller thresholds and therefore reflected routine clinical interpretation, we additionally performed an exploratory multivariable linear regression analysis with ESR (mm/hr) specified as a continuous dependent variable and CRP (mg/L), hemoglobin (g/dL), age, and sex included as predictors. This supplementary analysis allowed evaluation of associations across the full biological spectrum while preserving threshold-based modeling as the primary inferential framework.

Ethical considerations

The study was conducted in accordance with the Declaration of Helsinki. The study protocol was approved by the Institutional Ethics Committee of the participating institution (IEC-HR approval No. AIMS/IEC-HR/2025/87). The requirement for informed consent was waived due to the retrospective and anonymized nature of data collection.

## Results

Baseline characteristics

The final analytical cohort comprised 152 patients, of whom 133 (87.5%) were female. Seropositive rheumatoid arthritis (SPRA) accounted for 115 cases (75.7%), while 37 patients (24.3%) were classified as seronegative rheumatoid arthritis (SNRA).

The extent of missing laboratory data is summarized in Table 1. Hemoglobin values were missing in 1 patient (0.7%), erythrocyte sedimentation rate (ESR) in 22 patients (14.5%), C-reactive protein (CRP) in 46 patients (30.3%), and mean corpuscular volume (MCV) in 62 patients (40.8%).

**Table 1:** Extent of Missing Laboratory Data Extent of missing values for selected laboratory parameters in the analytical cohort. Data are presented as N (%). ESR: erythrocyte sedimentation rate; CRP: C-reactive protein; MCV: mean corpuscular volume; Hb: hemoglobin.

Laboratory Variable	Missing Values (n)	Percentage Missing (%)
Hemoglobin (Hb)	1	0.7%
ESR	22	14.5%
CRP	46	30.3%
MCV	62	40.8%

Among patients with available hemoglobin data, anemia was present in 89 of 151 individuals (58.9%). ESR positivity was observed in 97 of 130 patients (74.6%), and CRP positivity in 28 of 106 patients (26.4%).

Univariate comparisons by serostatus

In univariate analyses, SPRA patients demonstrated a more inflammatory laboratory profile. Anemia was more frequent in SPRA (74/114, 64.9%) than in SNRA (15/37, 40.5%; p=0.009). Similarly, ESR positivity was higher in SPRA (79/101, 78.2%) compared with SNRA (18/29, 62.1%; p=0.011). CRP positivity showed a comparable distribution (25/80, 31.3% vs. 3/26, 11.5%; p=0.013).

Baseline categorical characteristics stratified by serostatus are summarized in Table 2. Age, summarized as mean ± standard deviation (SD), is presented in Table 3, and quantitative laboratory variables, expressed as median (interquartile range), are presented in Table 4.

**Table 2:** Baseline Categorical Characteristics by Serostatus Comparison of categorical clinical and laboratory characteristics between seropositive rheumatoid arthritis (SPRA) and seronegative rheumatoid arthritis (SNRA). Data are presented as N (%) unless otherwise indicated. RF and anti-CCP positivity were defined according to predefined laboratory thresholds as described in the Methods section. Denominators reflect available case data where applicable. SPRA: seropositive rheumatoid arthritis; SNRA: seronegative rheumatoid arthritis; RF: rheumatoid factor; anti-CCP: anti-cyclic citrullinated peptide; ESR: erythrocyte sedimentation rate; CRP: C-reactive protein.

Characteristic	Overall (N=152)	SPRA (n=115)	SNRA (n=37)	p-value
Female sex, n (%)	133 (87.5%)	105 (91.3%)	28 (75.7%)	0.020
RF positive, n (%)	103 (67.8%)	103 (89.6%)	0 (0%)	<0.001
Anti-CCP positive, n (%)	98 (64.5%)	98 (85.2%)	0 (0%)	<0.001
Anemia present, n (%)	89/151 (58.9%)	74/114 (64.9%)	15/37 (40.5%)	0.009
ESR positive, n (%)	97/130 (74.6%)	79/101 (78.2%)	18/29 (62.1%)	0.011
CRP positive, n (%)	28/106 (26.4%)	25/80 (31.3%)	3/26 (11.5%)	0.013

**Table 3:** Baseline Continuous Variables Presented as Mean ± Standard Deviation Comparison of normally distributed continuous variables between SPRA and SNRA groups. Data are presented as mean ± standard deviation (SD). SPRA: seropositive rheumatoid arthritis; SNRA: seronegative rheumatoid arthritis.

Variable	Overall (N=152)	SPRA (n=115)	SNRA (n=37)	p-value
Age (years)	47.8 ± 12.1	47.7 ± 12.1	48.6 ± 12.3	0.718

**Table 4:** Baseline Continuous Variables Presented as Median (Interquartile Range) Comparison of non-normally distributed continuous variables between SPRA and SNRA groups. Data are presented as median (interquartile range [IQR]). SPRA: seropositive rheumatoid arthritis; SNRA: seronegative rheumatoid arthritis; RF: rheumatoid factor; anti-CCP: anti-cyclic citrullinated peptide; Hb: hemoglobin.

Variable	Overall (N=152)	SPRA (n=115)	SNRA (n=37)	p-value
RF (IU/mL), Median	45.0 [1.0–151.6]	73.1 [24.7–200.0]	0.0 [0.0–7.9]	<0.001
anti-CCP (U/mL), Median	157.1 [0.5–200.0]	195.0 [51.2–500.0]	0.0 [0.0–1.5]	<0.001
Hb (g/dL), Median	11.1 [10.3–12.2]	10.9 [10.1–12.0]	11.6 [10.9–12.5]	0.015

Correlation analysis

**Figure 2:**
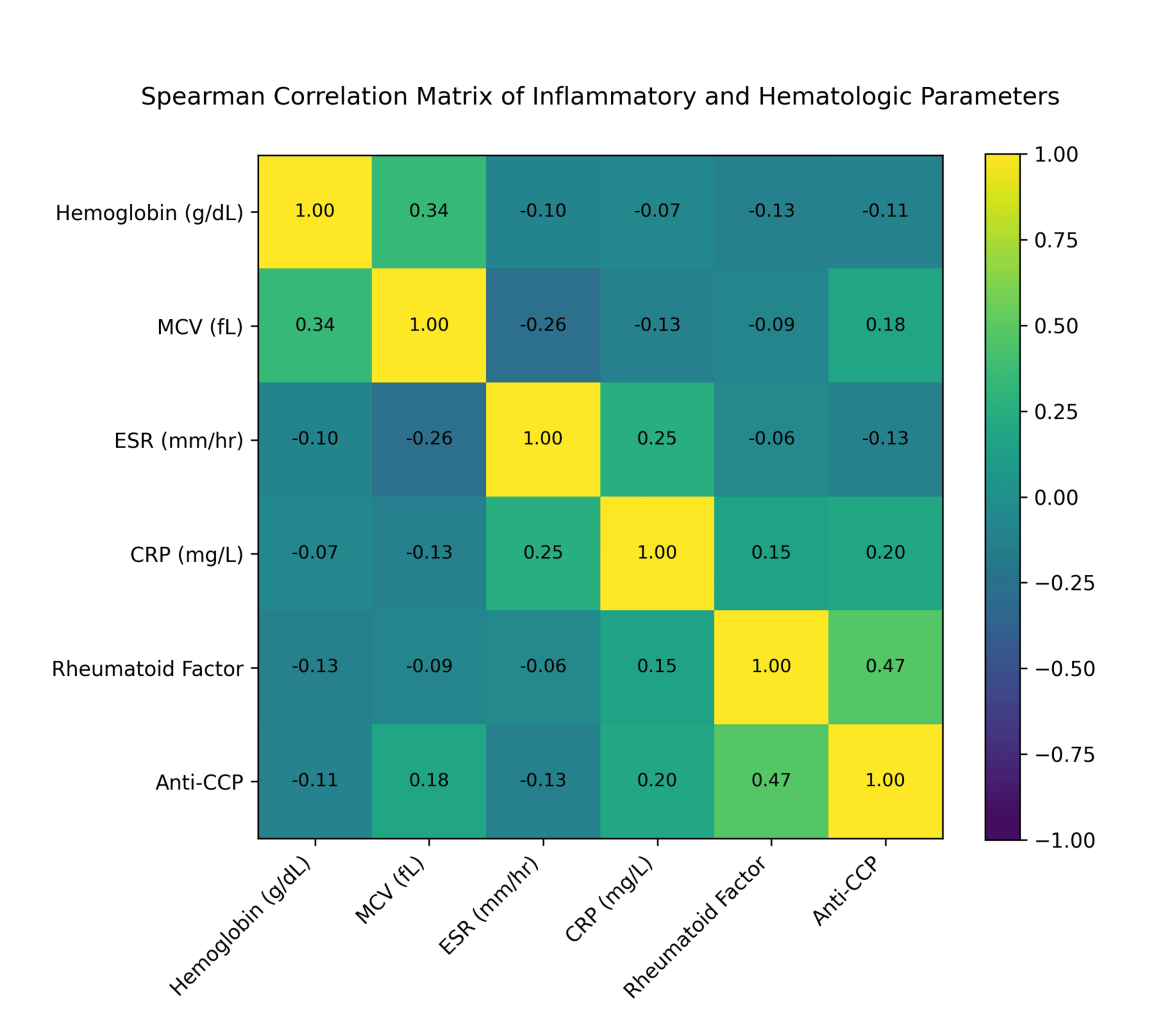
Spearman Correlation Matrix of Inflammatory and Hematologic Parameters Heatmap illustrating Spearman rank correlations among hemoglobin, mean corpuscular volume (MCV), erythrocyte sedimentation rate (ESR), C-reactive protein (CRP), rheumatoid factor (RF), and anti-cyclic citrullinated peptide (anti-CCP). Correlation coefficients are displayed within each cell. ESR: erythrocyte sedimentation rate; CRP: C-reactive protein; RF: rheumatoid factor; anti-CCP: anti-cyclic citrullinated peptide; MCV: mean corpuscular volume.

The Spearman rank correlation heatmap (Figure 2) demonstrated positive correlations between autoantibody titers (rheumatoid factor and anti-cyclic citrullinated peptide) and inflammatory markers (ESR and CRP). Hemoglobin showed a weak inverse correlation with ESR. These interrelationships supported the use of multivariate modeling to evaluate independent associations.

Multivariate logistic regression

**Figure 3:**
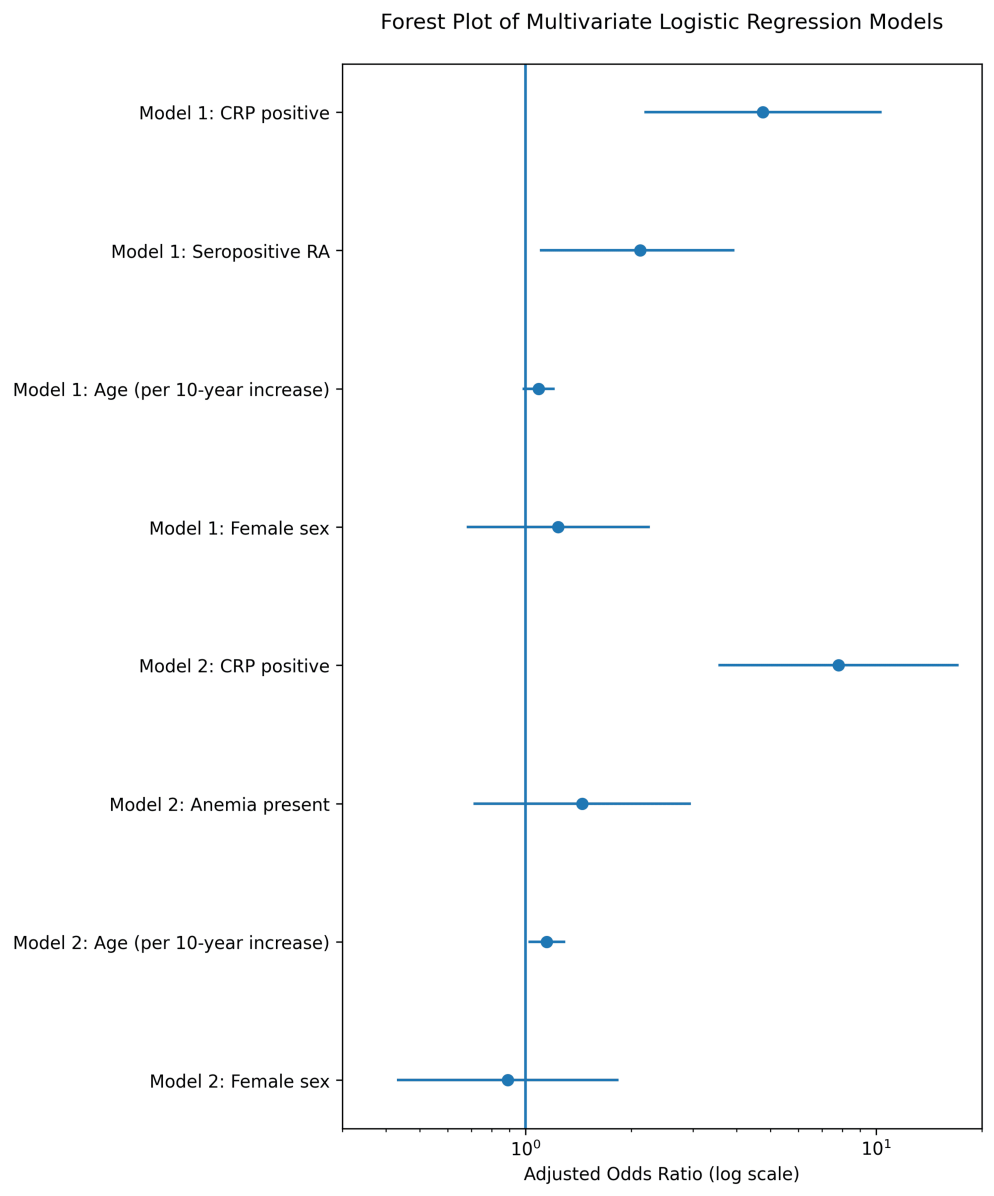
Forest Plot of Multivariate Logistic Regression Models Forest plot illustrating adjusted odds ratios (aORs) and 95% confidence intervals (CIs) from multivariate logistic regression analyses. Model 1 evaluated independent predictors of anemia. Model 2 evaluated independent predictors of ESR positivity. Analyses were based on pooled estimates from 20 multiply imputed datasets using Rubin’s rules. The vertical reference line represents an odds ratio of 1. aOR: adjusted odds ratio; CI: confidence interval; ESR: erythrocyte sedimentation rate; CRP: C-reactive protein.

Two multivariate logistic regression models were constructed to evaluate independent predictors of anemia and ESR positivity using pooled estimates from 20 multiply imputed datasets (Table 5). The corresponding adjusted odds ratios are visually summarized in Figure 3.

**Table 5:** Multivariate Logistic Regression Models Predicting Anemia and ESR Positivity Multivariate logistic regression analyses evaluating independent predictors of anemia (Model 1) and ESR positivity (Model 2). Adjusted odds ratios (aORs) and 95% confidence intervals (CIs) are derived from pooled estimates across 20 multiply imputed datasets using Rubin’s rules. Model 1 included CRP positivity, serostatus, age (per 10-year increase), and sex as predictors of anemia. Model 2 included CRP positivity, anemia status, age (per 10-year increase), and sex as predictors of ESR positivity. aOR: adjusted odds ratio; CI: confidence interval; ESR: erythrocyte sedimentation rate; CRP: C-reactive protein.

Predictor	Adjusted Odds Ratio (aOR)	95% Confidence Interval	p-value
Model 1: Predictors of Anemia			
CRP positive	4.75	2.18–10.36	<0.001
Seropositive rheumatoid arthritis	2.12	1.10–3.94	0.024
Age (per 10-year increase)	1.09	0.98–1.21	0.112
Female sex	1.24	0.68–2.26	0.480
Model 2: Predictors of ESR Positivity			
CRP positive	7.81	3.55–17.18	<0.001
Anemia present	1.45	0.71–2.96	0.305
Age (per 10-year increase)	1.15	1.02–1.30	0.021
Female sex	0.89	0.43–1.84	0.756

In Model 1, CRP positivity was the strongest independent predictor of anemia (adjusted odds ratio [aOR] 4.75; 95% confidence interval [CI] 2.18-10.36; p<0.001). Seropositive rheumatoid arthritis remained independently associated with anemia (aOR 2.12; 95% CI 1.10-3.94; p=0.024), while age and sex were not statistically significant predictors.

In Model 2, CRP positivity demonstrated a strong independent association with ESR elevation (aOR 7.81; 95% CI 3.55-17.18; p<0.001). Anemia was not independently associated with ESR positivity after adjustment (aOR 1.45; 95% CI 0.71-2.96; p=0.305). Increasing age showed a modest association (aOR 1.15 per 10-year increase; 95% CI 1.02-1.30; p=0.021), whereas sex was not statistically significant.

Sensitivity analyses restricted to complete-case datasets demonstrated consistent direction and magnitude of associations. Given the known limitations of complete-case restriction when data are not missing completely at random, multiply imputed estimates were retained as the primary analytic results.

Morphologic anemia patterns

Among 89 anemic patients, 87 had available mean corpuscular volume (MCV) data and were classified morphologically as 47 (54.0%) normocytic and 40 (46.0%) microcytic, while two patients had missing MCV values. No macrocytic anemia was identified.

Comparisons across non-anemic (n=62), microcytic (n=40), and normocytic (n=47) groups are shown in Table 6. Median ESR differed significantly across groups, with the highest values observed in the normocytic subgroup (62.0 mm/hr [IQR 45.0-84.0]; p<0.001). Median CRP values demonstrated a similar gradient across groups (p<0.001). The frequency of SPRA also varied significantly among anemia subtypes (p=0.019).

**Table 6:** Inflammatory Profiles by Morphologic Anemia Subtype Comparison of inflammatory markers and serostatus across non-anemic, microcytic anemia, and normocytic anemia groups. Data are presented as median (interquartile range [IQR]) or N (%). Denominators reflect cases with available data. One patient was missing hemoglobin data, and two anemic patients were missing MCV values; therefore, a total of 149 patients are categorized in this table. ESR: erythrocyte sedimentation rate; CRP: C-reactive protein; SPRA: seropositive rheumatoid arthritis; MCV: mean corpuscular volume.

Parameter	Non-anemic (n=62)	Microcytic anemia (n=40)	Normocytic anemia (n=47)	p-value
ESR (mm/hr), Median	35.0 [24.0–48.0]	39.0 [28.0–55.0]	62.0 [45.0–84.0]	<0.001
CRP (mg/L), Median	5.6 [1.0–10.2]	8.1 [1.0–13.0]	38.5 [11.6–74.9]	<0.001
SPRA frequency, n (%)	40/62 (64.5%)	29/40 (72.5%)	41/47 (87.2%)	0.019

These findings highlight heterogeneity in morphologic anemia patterns and their differential association with inflammatory burden.

Continuous modeling of ESR

In exploratory continuous modeling, CRP demonstrated a positive quantitative association with ESR, whereas hemoglobin showed minimal independent contribution. However, the overall explanatory power of the linear model was modest, reinforcing the clinical utility of threshold-based interpretation in routine practice.

## Discussion

This multivariate analysis demonstrates that in this North Indian rheumatoid arthritis cohort, CRP-defined systemic inflammation demonstrated the strongest independent association with ESR elevation within the multivariable framework applied. Although anemia is known to influence sedimentation dynamics, its effect was not independently significant after adjustment for inflammatory burden. These findings suggest that, within this dataset, ESR elevation more closely reflects biochemical inflammation than hematologic variation alone. Elevated ESR values were also observed among non-anemic patients, further supporting the interpretation that inflammatory activity rather than hemoglobin variation primarily explained ESR elevation in this cohort.

From a mechanistic perspective, ESR is influenced by erythrocyte aggregation, which is driven primarily by acute-phase reactants such as fibrinogen and immunoglobulins. In inflammatory states, increased plasma protein concentrations promote rouleaux formation and accelerate sedimentation. These observations are consistent with established physiological models of sedimentation and prior literature describing the influence of acute-phase proteins on ESR independent of red cell mass [22,23]. While reduced hemoglobin concentration may theoretically modify sedimentation by altering red blood cell mass and plasma viscosity, the multivariate findings in this cohort indicate that inflammatory signaling exerts a quantitatively stronger influence on ESR values. Clinically, this supports interpreting ESR in conjunction with CRP and overall clinical assessment rather than attributing elevation solely to hematologic factors.

An additional important observation was the independent association between seropositive rheumatoid arthritis (SPRA) and anemia after adjustment for CRP. This finding suggests that antibody-positive disease may represent a phenotype characterized by greater hematologic involvement beyond measured systemic inflammation alone. Prior literature has linked seropositivity to higher disease burden and altered cytokine activity, which may contribute to inflammation-associated changes in iron metabolism and erythropoiesis [12]. However, these results represent statistical associations within a cross-sectional framework and should not be interpreted as evidence of causality.

The analysis also demonstrated heterogeneity in morphologic anemia patterns. Normocytic anemia was associated with significantly higher ESR and CRP values compared with both microcytic and non-anemic groups, whereas inflammatory marker levels in the microcytic subgroup were comparatively lower. These findings are broadly consistent with prior Indian cohorts reporting substantial contributions from both inflammation-related and nutritional mechanisms to anemia burden in RA [11]. However, given the absence of iron studies, the present classification is morphologic rather than etiological. Overlap between inflammation-associated anemia and iron-deficiency anemia is well recognized, and mean corpuscular volume alone cannot definitively differentiate these entities [10].

Although differences in inflammatory profiles were observed, this study was not designed to assess management outcomes or long-term prognostic implications. Emerging literature suggests that anemia subtypes may demonstrate differing associations with mortality and cardiovascular outcomes in RA [24,25], but the cross-sectional nature of the present dataset precludes such inference. The relatively balanced distribution of morphologic anemia patterns in this cohort underscores the complexity of hematologic manifestations in RA and highlights the need for prospective evaluation incorporating iron parameters and longitudinal follow-up.

The overall laboratory profile, including generally preserved renal and hepatic function, suggests that the cohort was representative of patients typically managed in tertiary rheumatology practice within the region [26-28].

Taken together, these findings support the continued use of ESR as part of an integrated inflammatory assessment in rheumatoid arthritis, even in settings where anemia prevalence is high, provided results are interpreted within a combined clinical and laboratory framework.

Limitations

This study has several limitations. The retrospective single-center design may limit generalizability. Laboratory acquisition was determined by clinical indication rather than a standardized research protocol, resulting in incomplete availability of certain parameters, particularly CRP and MCV. Anemia classification relied on mean corpuscular volume in the absence of iron indices; while morphologic categorization is commonly used in clinical practice, it does not establish definitive etiologic diagnoses and may not fully account for mixed anemia states [10]. Missing laboratory data were addressed using multiple imputation in accordance with established epidemiologic methodology [29,30]; while this approach preserves statistical power and reduces bias compared with complete-case restriction, it relies on the assumption that data are missing at random. Standardized disease activity measures such as DAS28 were not uniformly available, limiting the correlation of laboratory findings with validated composite activity scores. The study design was cross-sectional and based on a single baseline laboratory panel per patient, precluding assessment of longitudinal inflammatory dynamics. Although exploratory continuous modeling was performed, threshold-based interpretation aligned with age- and sex-adjusted Miller criteria was retained to reflect routine clinical practice. Findings should therefore be interpreted within the broader clinical and laboratory context.

## Conclusions

In this North Indian rheumatoid arthritis cohort, C-reactive protein-defined inflammatory activity demonstrated the strongest independent association with erythrocyte sedimentation rate elevation after multivariable adjustment, whereas anemia did not independently drive elevation above age- and sex-adjusted thresholds. Normocytic and microcytic anemia patterns coexisted and were associated with differing inflammatory profiles, underscoring the heterogeneity of hematologic manifestations in rheumatoid arthritis. Within the limitations of this cross-sectional design, these findings support an integrated interpretation of ESR alongside complementary laboratory parameters and warrant prospective studies incorporating detailed iron indices and standardized disease activity measures.
